# Interruption in physical activity bout analysis: an accelerometry research issue

**DOI:** 10.1186/1756-0500-7-284

**Published:** 2014-05-06

**Authors:** Makoto Ayabe, Hideaki Kumahara, Kazuhiro Morimura, Hiroaki Tanaka

**Affiliations:** 1 Faculty of Computer Science and Systems Engineering, Okayama Prefectural University, 111 Kuboki, Soja, Okayama 7191197, Japan; 2 Faculty of Health and Sports Science, Fukuoka University, 8-19-1 Nanauma, Jhonan, Fukuoka 8140180, Japan; 3 Faculty of Nutritional Sciences, Nakamura Gakuen University, 5-7-1 Befu, Jhonan, Fukuoka 8140104, Japan

**Keywords:** Accelerometer, Pedometer, Pedometry, Methodology, Accuracy, Validity

## Abstract

**Background:**

The purpose of the present investigation was to clarify the impact of the treatment of interruptions on the durations and the frequency of the physical activity (PA) bouts under free-living conditions.

**Methods:**

One hundred and forty adults (50 ± 7 years) wore an accelerometer (Lifecorder) for seven consecutive days under free-living conditions. According to the minutes by minutes metabolic equivalents (METs) value, the PA was divided into one of three intensity categories: light intensity PA (LPA, < 3 METs), moderate intensity PA (MPA, 3 to 6 METs), vigorous intensity PA (VPA, > 6 METs), and the sum of the MPA and VPA was defined as moderate to vigorous intensity PA (MVPA, > 3 METs). Thereafter, based on the time series data, we defined MVPA bouts as PA that was maintained at no less than 3 METs completely for 10 minutes or longer with or without allowing for a one-minute or a two-minute break (<3METs).

**Results:**

The frequency and duration of the continuous MVPA bouts lasting longer than 10-min were significantly lower and shorter compared with that in the non-continuous MVPA bouts allowing a one- or two-minute interruption (4.11 ± 1.65, 6.58 ± 2.72 and 8.97 ± 3.55 bouts/day, 71.62 ± 33.66, 119.03 ± 49.35 and 169.75 ± 65.87 min/day, P < 0.05). The number of days with a total time of MVPA bouts of 30-min was significantly lower in the continuous MVPA bouts compared with that in the non-continuous MVPA bouts allowing a 1-min or 2-min interruption (5.36 ± 1.65, 6.39 ± 1.07 and 6.65 ± 0.85 days/week).

**Conclusion:**

The treatment of interruptions for the setting of the accelerometer affects the estimation of the MVPA bouts under free-living conditions in middle-aged to older adults. The best analysis process with regard to the accelerometer quantifying the break to reflect the real behavioral pattern and the physiological stress in such subjects remains unclear.

## Background

The American College of Sports Medicine and the American Heart Association recommend that all healthy adults 18 to 65 years of age engage in moderate-intensity aerobic (endurance) physical activity (MPA) or vigorous-intensity aerobic physical activity (VPA) in order to promote and maintain health [1]. Since several consensus statements also recommend engaging in moderate to vigorous intensity physical activity (MVPA) [2,3], spending a longer time performing MVPA is widely accepted to be a goal for a healthy lifestyle.

Additionally, moderate-intensity aerobic activity, which is generally equivalent to a brisk walk that noticeably accelerates the heart rate, can count toward the 30 minute minimum when bouts of activity lasting 10 minutes or more are performed [1]. A previous investigation suggested that bouts of physical activity lasting ≥10 minutes might be a more time-efficient strategy to the decrease body mass index and waist circumference [4]. Furthermore, Catenacci et al., suggested that sustained volitional activity (i.e., ≥ 10 min in duration) might play an important role in the long-term maintenance of weight loss [5]. These findings clearly indicated that the accumulation of MVPA bouts lasting longer than 10-min would be a beneficial strategy to obtain health benefits.

Therefore, the accurate assessment of the frequency and duration of the MVPA bouts has been an important research issue. An accelerometer is one of the reliable assessments of MVPA bouts under free-living conditions, and there are several criteria for determining MVPA bouts lasting longer than 10-min [6-11]. Based on the principals of the accelerometer features, the epoch length and the treatment of the interruptions (breaks during exercise) have important roles in determining the frequency and durations of MVPA bouts. With regard to the epoch length, recent studies consistently showed that the frequency and duration of the MVPA were apparently higher under the longer epoch setting compared with that under the shorter epoch setting [6,7].

However, with regard to the treatment of the interruptions, to the best of our knowledge, only two studies have quantitatively examined the effects of the treatment of the interruptions on the frequency and duration of MVPA bouts [8,9]. Masse et al [9] suggested that the algorithm comparisons also showed that MVPA bouts did not differ significantly when all algorithms allowed a 1- or 2-min interruption anywhere in the bout; however, when no interruption was allowed, the number of minutes of MVPA bouts decreased significantly. In contrast, Miller et al. did not find the significant differences in bout duration (min) of PA at >3METs lasting longer than 10-min among 3 different interruption criteria, whereas the number of bouts for ≥10-min and ≥3 METS was greater when a 1- or 2-min interruption was allowed. These studies consistently indicated that the treatment of the interruption affects the accelerometer, however, in regard to the “min of MVPA”, the results have been conflicting. Since the min of MVPA is import index of MVPA as same as “intensity of MVPA” and/or “frequency of MVPA”, the treatment of the interruption has been left as one of the important research issue.

Since there were unexpectedly wide variations in the MVPA bouts depending on how the interruptions were treated [5,12-15], the impact of the treatment of interruptions on the estimation of the MVPA bouts should be clarified. Thus, the purpose of the present investigation was to clarify the impact of the treatment of interruptions on the durations and the frequency of the MVPA bouts recorded by an accelerometer.

## Methods

### Subjects

One hundred and forty adults, aged 32 to 72 years old, participated in the present investigation. Most of subjects are same as the previous publications [6], the details are not shown in the present paper. The characteristics of the participants are shown in Table 1. After an explanation of the study requirements, each subject read and signed a consent form. The ethics committee of Fukuoka University approved all procedures used in the present investigation. All procedures related to the measurements were performed between March and April.

**Table 1:** Characteristics of the participants

	**All**	**Female**	**Male**
N	140	45	95
Age (years)	50 ± 7	50 ± 7	50 ± 8
Height (cm)	164.0 ± 14.5	153.3 ± 20.5	169.1 ± 5.8*
Body weight (kg)	66.6 ± 12.4	54.7 ± 8.4	72.2 ± 9.8*

Data are expressed as the means with the standard deviation (mean ± SD).

*Significantly different between males and females (p < 0.01).

### Physical activity assessments

The use of an accelerometer and the procedure for wearing an accelerometer are same as the previous publications [6], the present manuscript document it briefly. During the course of the present investigation, all participants wore a pedometer with a uni-axial accelerometer (Lifecorder Ex 4-sec version, Kenz, Nagoya, Japan; LC) under free-living conditions [16-18]. After reading the instructions regarding the general care of these activity monitors, the participants wore the LC for 10 days continuously, except while sleeping or bathing. The LC was placed on the left anterior mid-line of the thigh on the waist band of the participant’s clothing. After the data collection period was completed, the participants returned the LC by mail to the investigators.

### The analysis of the accelerometer data

**Figure 1:**
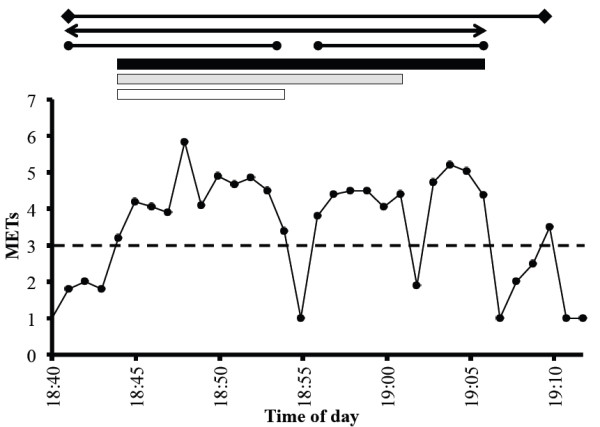
**Typical data treatment for the physical activity bouts determination by different criteria.** The illustration shows the MET values determined by an accelerometer from 18:40 to 19:10 in one of the participants (Sub #1). The boxes and lines above the illustrations indicating the physical activity bouts determined by the different criteria. The white box means physical activity > 3 METs lasting > 10-min without an interruption (10-min), the gray box means physical activity > 3 METs lasting > 10-min allowing a 1-min interruption (17-min), and the black box means physical activity > 3 METs lasting > 10-min allowing a 2-min interruption (22-min). The black lines sandwiched by the closed circle means physical activity lasting > 10-min without an interruption (14-min and 11-min), the black line sandwiched by the arrows means physical activity lasting > 10-min allowing a 1-min interruption (26-min), and the black line sandwiched by the squares means physical activity lasting > 10-min allowing a 2-min interruption (30-min).

The stored data could be uploaded to a personal computer for the analysis. To be included in the analysis, each participant had to have at least seven days when they sufficiently wore the device (>10 hours) [19]. The intensity categories, determined every four seconds, were converted to METs values based on the equation developed by the previous investigation [16]. Thereafter, the METs values were averaged for every 60 seconds. The epoch length was set at 60 sec in the present investigation according to the previous investigations [8,9]. According to the minutes by minutes METs values, the PA was divided into one of three intensity categories: light intensity PA (LPA, < 3 METs), moderate intensity PA (MPA, 3 to 6 METs) or vigorous intensity PA (VPA, > 6 METs), and the sum of the MPA and VPA was defined as moderate to vigorous intensity PA (MVPA, > 3 METs). Thereafter, based on the time series data, we defined PA bouts as PA that was maintained at no less than 1.5 METs for 10 minutes or longer, and a MVPA bout was defined as PA that was maintained at no less than 3 METs for 10 minutes or longer. Furthermore, PA bouts including the interruptions were defined as PA that was maintained at no less than 1.5 METs for 10 minutes or longer, allowing for a one-minute or a two-minute break (one column or two columns under 1.5 METs), PA that was maintained at no less than 3 METs for 10 minutes or longer allowing for a one-minute or a two-minute break (one column or two columns under 3 METs) (Figure 1).

All accelerometer outputs were averaged over seven days. Since the present investigation was focused on the accelerometer’s algorithms, the inter-individual variability, such as the day-to-day variability or hourly variability, were not evaluated, according to the findings of previous validation studies [6,18].

Statistics. The data were expressed as the means with standard deviations. The potential gender-related differences in the obtained data were analyzed by the unpaired *t*-test. A one-way ANOVA and Scheffe’s test were used to compare the PA levels for the three different treatment of interruptions (none, one-minute, two minute). The correlations among MVPA bouts determined by three different criteria were analyzed by Pearson’s R. Statistical significance was set at P < 0.05. All statistical analyses were performed using the StatView software program (version 5.0.1, SAS Institute, Cary, NC, USA).

## Results

The height and body weight were significantly different between males and females (p < 0.01, Table 1). The time spent in sporadic PA is shown in Table 2. None of the accelerometer outputs significantly differed between genders.

**Table 2:** The time spent in sporadic light, moderate and vigorous intensity physical activity determined by an accelerometer using one-minute epoch length

	**All**	**Female**	**Male**
Light intensity physical activity (min/day)	197 ± 63	233 ± 73	179 ± 49
Moderate intensity physical activity (min/day)	34 ± 20	29 ± 16	36 ± 21
Vigorous intensity physical activity (min/day)	0.83 ± 2.47	1.07 ± 2.09	0.72 ± 2.63
Moderate to vigorous intensity physical activity (min/day)	34 ± 21	30 ± 17	37 ± 22

Data are expressed as the means with the standard deviation (mean ± SD).

Table 3 shows the time and the frequency for the PA bouts as determined using the three different criteria. The frequency and duration of the continuous activity bouts lasting longer than 10-min were significantly lower and shorter compared with that in the non-continuous PA bouts allowing a one-minute or two-minute interruption (P < 0.05). Additionally, the frequency and duration of the PA bouts significantly differed within the two non-continuous settings (p < 0.05). Similarly, with regard to the MVPA bouts determinations, both the frequency and duration were significantly differ among the three criteria settings. Furthermore, the numbers of days where the subjects participated in one or more MVPA bouts and the numbers of days where the total time of MVPA bouts reached 30 minutes, were significantly lower in the continuous MVPA bouts compared with that in the non-continuous MVPA bouts allowing a one-minute or two-minute interruption (P < 0.05).

**Table 3:** Comparison of the physical activity lasting longer than 10 minutes allowing no breaks, a one-minute break or a two-minute break

		**Continuous activity bouts**	**Non-continuous activity bouts allowing a 1-min interruption**	**Non-continuous activity bouts allowing a 2-min interruption**
PA lasting ≧10-min	Number of bouts (bouts/day)	4.11±1.65	6.58±2.72**	8.97±3.55**,‡
	Daily duration (min/day)	71.62±33.66	119.03±49.35**	169.75±65.87**,‡
	Number of days with at least one bout (days/week)	6.50±1.00	6.71±0.83*	6.79±0.78**
	Number of days accumulating ≧30 min (days/week)	5.36±1.65	6.39±1.07**	6.65±0.85**
				
MVPA lasting ≧10-min	Number of bouts (bouts/day)	0.60±0.68	0.82±0.83*	1.02±0.88**,†
	Daily duration (min/day)	11.43±15.23	16.38±19.57*	21.28±22.37**,†
	Number of days with at least one bout (days/week)	2.49±2.22	2.96±2.27	3.47±2.17**
	Number of days accumulating ≧30 min (days/week)	1.01±1.64	1.49±1.95*	1.90±2.10**

Data are expressed as the means with the standard deviation (mean±SD).

PA; Physical activity, MVPA; moderate to vigorous intensity physical activity. *, **Significantly different compared with the Continuous activity bouts (*p<0.05, **p<0.01). †, ‡Significantly different compared with the Non-continuous activity bouts allowing a 1-min interruption (†p<0.05, ‡p<0.01).

**Figure 2:**
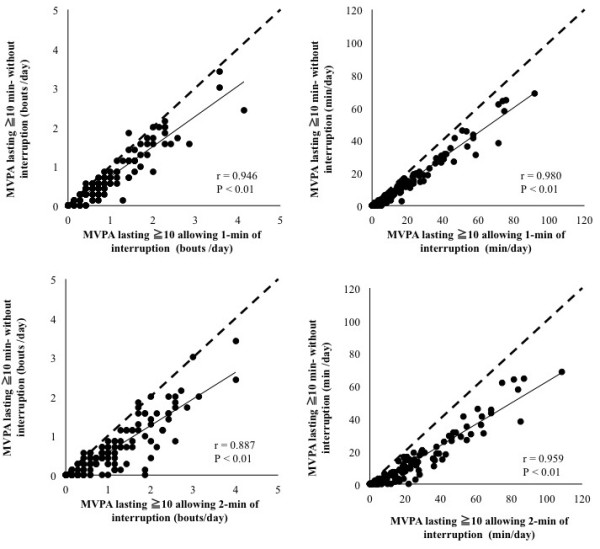
**The relationships between moderate to vigorous intensity physical activity lasting longer than ten minutes determined by the three criteria.** The dashed lines indicate the identity line (y = x).

Figure 2 shows the relationship between the continuous MVPA bouts and the non-continuous MVPA bouts including a one- or two-minute break (Figure 2). The frequency and duration of the continuous MVPA bouts was significantly associated with the non-continuous MVPA bouts including a one- or two-minute break (r = 0.887 – 0.980, P < 0.01).

## Discussion

The present investigation examined the role of treatment of the interruptions in activity on the evaluation of PA bouts with regard to accelerometer-based PA assessment in middle- to older-aged individuals. The main finding of the present investigation is that the frequency and duration of the PA bouts were significantly lower and shorter when a one- or two-minute interruption was allowed, compared with that when no interruptions were allowed (Table 3), whereas the frequency and duration of the continuous MVPA bouts was significantly associated with the non-continuous MVPA bouts including a one- or two-minute break. As a result, the number of days accumulating at least 30-min of MVPA bouts allowing a one- or two-minute interruption was significantly higher compared with the number of days accumulating at least 30-min of continuous MVPA bouts without an interruption. Although several studies examined the effects of the treatment of interruptions on the accelerometer’s out puts [8,9], the present investigation firstly showed that both of the daily frequency and the daily duration of MVPA bouts significantly differed according to the choice of the treatment of interruptions. Furthermore the frequency and duration of MVPA bouts gradually increased from when allowing no interruptions to when including 1-min or 2-min interruptions, additionally these significant difference were also found between that when allowing 1-min or 2-min interruptions and when allowing 2-min interruptions. These results indicate that, in the accelerometer’s outputs analysis, the treatment of the interruptions affects the estimation of the MVPA bouts under free-living conditions in middle-aged to older adults. Both the frequency and the duration of the MVPA bouts would be significantly higher depending on the allowing duration of interruptions. Thus, caution should be exercised regarding the accelerometer’s data setting when discussing the MVPA bouts reported in previous publications.

One of the original findings of the present investigation is that, with regard to the accelerometer-based MVPA assessment, allowing one- or two-minute interruptions results in a larger number of MVPA bouts compared with that when not allowing any interruptions under free-living conditions in middle aged to older adults. These findings are supported by the previous findings, where two studies demonstrated that allowing short interruptions increased the frequency of the MVPA bouts compared with that not allowing any interruptions [8,9]. Miller et al. reported that the frequency of MVPA bouts was 0.5 ± 0.8 bouts/day for the continuous MVPA bouts, 0.6 ± 0.8 bouts/day for the non-continuous MVPA bouts allowing a one-minute interruption and 0.6 ± 0.9 bouts/day for the non-continuous MVPA bouts allowing a two-minute interruption [8]. Similarly, Masse al. reported that the frequency of MVPA bouts was 3.0 ± 6.8 bouts/day, 4.1 ± 7.8 bouts/day and 4.9 ± 7.9 bouts/day for the same measurements [9]. In the present investigation, the frequency of the MVPA bouts differed from 0.60 ± 0.68 to 1.02 ± 0.88 bouts/day. Based on the these findings, a range of 0.1 to 1.9 bouts/day of MVPA might be detected due to the treatment of the interruptions, and these differences resulting from the different criteria would be increased based on whether a one-minute or two-minute interruption was allowed. As a result, the duration of MVPA bouts allowing a two-minute break (21.28 ± 22.37 min/day) was almost double the duration of MVPA bouts without interruptions (11.43 ± 15.23 min/day). Furthermore, the duration of the MVPA bouts allowing a one-minute break differed significantly compared with the MVPA bouts allowing a two-minute break, the same as was noted in previous investigations [9]. These finding clearly indicate that the treatment of the interruption has a significant impact on the estimation of MVPA bouts under free-living conditions, and these differences may be also found between the MVPA bouts allowing a one-minute break and the MVPA bouts allowing a two-minute break.

Based on these findings, the large variability in the reported MVPA bouts has likely been due to the choice of the treatment of the interruptions. The duration of MVPA bouts allowing a one-minute break was 25.8 ± 23.4 min/day in normal weight subjects [5]. Furthermore, the duration of MVPA bouts allowing a two-minute break was 9 ± 13 [20], 9.1 ± 0.5 and 6.6 ± 0.5 min/day in males and females from the NHANES 2003-2006 study [21], 13 to 16 min/day in Swedish adults and 6 to 10 min/day in US adults [14]. In contrast, the duration of the continuous MVPA not including interruptions has been much shorter. Davis et al reported that the continuous MVPA bouts lasting longer than 10-min were 0.4 to 0.6 min/day in British, Italian and French volunteers, whereas the duration of the sporadic MVPA was 19.9 to 39.4 min/day [15].

There is no doubt that the treatment of the interruption has a significant impact on the estimation of MVPA bouts under free-living conditions, because these differences are dependent on the data treatment process. For example, if individuals repeated a five-minute walking bout following a one minute break, the MVPA bouts would be described as lasting zero minutes, 11 minutes and 17 minutes when the analysis allows no interruptions, a one-minute interruption and a two-minute interruption, respectively. Furthermore, the usual habitual physical activity mainly consists of intermittent activities [22]. In developed countries, purposeful walking may be frequently stopped by traffic signals and/or crowds, so the METs value will be below 3 METs. Thus, the magnitude of the impact of the interruption treatment on the MVPA bout estimation would be increased in the individuals accumulating MVPA bouts from habitual PA (walking to work, shopping, gardening, etc.) rather than for the individuals participating in purposeful continuous PA (jogging, running, sports activities, etc.).

It should be noted that the present investigation does not indicate that not allowing an interruption is the best procedure for the MVPA bouts estimation. Masse et al. [9] suggested that it appeared reasonable to allow a one- or two-minute interruption anytime during the bout, because extracting MVPA bouts has been used to determine whether the participant met the current physical activity recommendations. We agree that this may be more reasonable.

However, in addition to the treatment of interruptions, the choice of the epoch length is an important contributor to the MVPA bouts estimation [6]. Furthermore, not only waist acceleration signals but also physiological stress, such as the oxygen uptake and/or heart rate response, and direct observation should be considered to define the optimum procedure for the data treatment of the accelerometer outputs for the MVPA bouts estimations.

There are several limitations associated with the present investigation that should be considered when interpreting the results. First, the type (uni-axial) and position (waist) of the accelerometer used in the present investigation does not allow for the collection of upper body activity, and thus may underestimate the total PA. Second, the participants evaluated in the present investigation were primarily middle-aged, non-active males and females. Furthermore, the participants all lived in urban areas, and buses and trains were their primary means of transportation.

## Conclusions

In summary, the present investigation examined the role of the treatment of the interruptions in the evaluation of PA bouts with regard to accelerometer-based PA assessment in middle- to older-aged individuals. The main finding of the present investigation is that, in the accelerometer’s output analysis, the treatment of the interruptions affects the estimation of the PA bouts under free-living conditions in middle-aged to older adults. Both the frequency and the duration of the MVPA bouts would be significantly higher when interruptions are allowed compared with that when not allowing any interruption. Thus, caution should be exercised regarding the treatment of the interruptions when discussing the MVPA bouts reported in previous publications. The optimal interruption treatment to estimate the MVPA bouts remains unclear, and will need to be assessed in future studies.

## Abbreviations

LC: Lifecorder; LPA: Light intensity physical activity; METs: Metabolic equivalents, MPA, Moderate intensity physical activity; MVPA: Moderate to vigorous intensity physical activity; PA: Physical activity; VPA: Vigorous intensity physical activity.

## Competing interests

None of the authors have any professional relationship with companies or manufacturers that will benefit from the results of the present study.

## Authors’ contributions

Planning: MA, HT, Conducting: MA, HK, KM, Data treatment: MA, KM, Reporting: MA, HK, HT. All authors read and approved the final manuscript.

## References

[B1] Haskell WL Lee IM Pate RR Powell KE Blair SN Franklin BA Macera CA Heath GW Thompson PD Bauman A Physical activity and public health: updated recommendation for adults from the American College of Sports Medicine and the American Heart Association Med Sci Sports Exerc 2007 39 1423 1434 10.1249/mss.0b013e3180616b27 17762377

[B2] Saris WH Blair SN van Baak MA Eaton SB Davies PS Di Pietro L Fogelholm M Rissanen A Schoeller D Swinburn B Tremblay A Westerterp KR Wyatt H How much physical activity is enough to prevent unhealthy weight gain? Outcome of the IASO 1st Stock Conference and consensus statement Obes Rev 2003 4 101 114 10.1046/j.1467-789X.2003.00101.x 12760445

[B3] Ishikawa-Takata K Tabata I Exercise and physical activity reference for health promotion 2006 (EPAR2006) J Epidemiol 2007 17 177 17827865 10.2188/jea.17.177 PMC7058479

[B4] Strath SJ Holleman RG Ronis DL Swartz AM Richardson CR Objective physical activity accumulation in bouts and nonbouts and relation to markers of obesity in US adults Prev Chronic Dis 2008 5 A131 18793519 PMC2578774

[B5] Catenacci VA Grunwald GK Ingebrigtsen JP Jakicic JM McDermott MD Phelan S Wing RR Hill JO Wyatt HR Physical Activity Patterns Using Accelerometry in the National Weight Control Registry Obesity (Silver Spring) 2011 6 1163 1170 21030947 10.1038/oby.2010.264 PMC4560526

[B6] Ayabe M Kumahara H Morimura K Tanaka H Epoch length and the physical activity bout analysis: An accelerometry research issue BMC Research Notes 2013 6 20 10.1186/1756-0500-6-20 23331772 PMC3558345

[B7] Dencker M Svensson J El-Naaman B Bugge A Andersen LB Importance of epoch length and registration time on accelerometer measurements in younger children J Sports Med Phys Fitness 2012 52 115 121 22525645

[B8] Miller GD Jakicic JM Rejeski WJ Whit-Glover MC Lang W Walkup MP Hodges ML Effect of Varying Accelerometry Criteria on Physical Activity: The Look AHEAD Study Obesity (Silver Spring) 2012 21 32 44 23505166 10.1038/oby.2012.118 PMC3430806

[B9] Masse LC Fuemmeler BF Anderson CB Matthews CE Trost SG Catellier DJ Treuth M Accelerometer data reduction: a comparison of four reduction algorithms on select outcome variables Med Sci Sports Exerc 2005 37 S544 S554 10.1249/01.mss.0000185674.09066.8a 16294117

[B10] Heil DP Brage S Rothney MP Modeling Physical Activity Outcomes from Wearable Monitors Med Sci Sports Exerc 2012 44 S50 S60 22157775 10.1249/MSS.0b013e3182399dcc

[B11] Tudor-Locke C Camhi SM Troiano RP A catalog of rules, variables, and definitions applied to accelerometer data in the national health and nutrition examination survey, 2003-2006 Prev Chronic Dis 2012 9 E113 22698174 10.5888/pcd9.110332 PMC3457743

[B12] Ayabe M Brubaker PH Sunami Y Musci R Kumahara H Schutz Y Tanaka H Objectively measured physical activity levels of Venetian adults J Sports Med Phys Fitness 2013 53 671 679 24247191

[B13] Ayabe M Aoki J Kumahara H Tanaka H Age-related differences in daily physical activity divided by bout duration: Preliminary findings in female convenience samples J Sports Sci 2012 30 709 713 10.1080/02640414.2012.667878 22401295

[B14] Hagstromer M Troiano RP Sjostrom M Berrigan D Levels and patterns of objectively assessed physical activity–a comparison between Sweden and the United States Am J Epidemiol 2010 171 1055 1064 10.1093/aje/kwq069 20406758

[B15] Davis MG Fox KR Physical activity patterns assessed by accelerometry in older people Eur J Appl Physiol 2007 100 581 589 10.1007/s00421-006-0320-8 17063361

[B16] Kumahara H Schutz Y Ayabe M Yoshioka M Yoshitake Y Shindo M Ishii K Tanaka H The use of uniaxial accelerometry for the assessment of physical-activity-related energy expenditure: a validation study against whole-body indirect calorimetry Br J Nutr 2004 91 235 243 10.1079/BJN20031033 14756909

[B17] Ayabe M Aoki J Ishii K Takayama K Tanaka H Pedometer accuracy during stair climbing and bench stepping exercises J Sports Sci Med 2008 7 249 254 24149457 PMC3761450

[B18] Ayabe M Ishii K Takayama K Aoki J Tanaka H Comparison of interdevice measurement difference of pedometers in younger and older adults Br J Sports Med 2010 44 95 99 10.1136/bjsm.2007.045179 18308892

[B19] Trost SG McIver KL Pate RR Conducting accelerometer-based activity assessments in field-based research Med Sci Sports Exerc 2005 37 S531 S543 10.1249/01.mss.0000185657.86065.98 16294116

[B20] Glazer NL Lyass A Esliger DW Blease SJ Freedson PS Massaro JM Murabito JM Vasan RS Sustained and Shorter Bouts of Physical Activity are Related to Cardiovascular Health Med Sci Sports Exerc 2012 45 109 115 22895372 10.1249/MSS.0b013e31826beae5 PMC4166425

[B21] Luke A Dugas LR Durazo-Arvizu RA Cao G Cooper RS Assessing Physical Activity and its Relationship to Cardiovascular Risk Factors: NHANES 2003-2006 BMC Public Health 2011 11 387 10.1186/1471-2458-11-387 21612597 PMC3123595

[B22] Orendurff MS Schoen JA Bernatz GC Segal AD Klute GK How humans walk: bout duration, steps per bout, and rest duration J Rehabil Res Dev 2008 45 1077 1089 10.1682/JRRD.2007.11.0197 19165696

